# Piloting of virtual group education for diabetes in Cape Town: An exploratory qualitative study

**DOI:** 10.4102/safp.v65i1.5635

**Published:** 2023-01-18

**Authors:** Robert J. Mash, Joleen Cairncross

**Affiliations:** 1 Division of Family Medicine and Primary Care, Faculty of Medicine and Health Sciences, Stellenbosch University, Cape Town, South Africa

**Keywords:** diabetes, patient education, counselling, primary care, behaviour change, telemedicine, virtual reality

## Abstract

**Background:**

Diabetes is a major public health problem. During the coronavirus disease 2019 (COVID-19) pandemic, patient education and counselling (PEC) for diabetes were curtailed. This project explored the possibility of offering group empowerment and training (GREAT) for diabetes via computer or tablets and Zoom video conferencing. The aim was to explore whether this was feasible in the low-income community context of primary health care in Cape Town, South Africa.

**Methods:**

Three dieticians facilitated four sessions of GREAT for diabetes with a group of five patients with type-2 diabetes. Once the programme was completed, focus group interviews were held with the facilitators and the patients to explore their experience. Interviews were recorded and analysed using a simplified framework method.

**Results:**

Usual primary care was not offering PEC and service delivery was brief and mechanistic. The content, resources and group processes were successfully translated into the virtual environment. The guiding style of communication was more difficult. Patients reported changes in their self-management and appreciated being able to save time and money while participating from home. Patients required considerable support and training to use the technology. All participants were concerned about safety and crime with the hardware.

**Conclusion:**

It was feasible to conduct GREAT for diabetes via tablets and Zoom video conferencing in this low-income community. To implement at scale, a number of concerns need to be addressed. The feasibility of conducting the sessions via smartphone technology should be evaluated.

**Contribution:**

Demonstrates how digital technology could be used to develop new ways of empowering people with type 2 diabetes.

## Introduction

Diabetes is the leading cause of death for women in South Africa, and the second highest cause of death in the whole population.^1^ It has become a major contributor to the burden of disease and a public health priority. During the coronavirus disease 2019 (COVID-19) pandemic, people with diabetes were more at risk of hospitalisation and death and this risk increased with worsening glycaemic control.^2^ At the same time, primary care facilities were re-organised to cope with the pandemic and reduce the risk of infection to people with comorbidities.^3^

In the public sector of Cape Town routine care for people with diabetes was reduced and in particular patient education and counselling (PEC) was mostly unavailable. Medication was delivered at home by community health workers and only those with emergencies or who were very uncontrolled attended the facilities.^4^

Prior to the COVID-19 pandemic, the need to improve PEC was recognised and Group Empowerment and Training (GREAT) for diabetes had been designed, developed and evaluated.^5^ A number of studies showed that GREAT for diabetes was cost-effective and improved both glycaemic and blood pressure control.^6,7,8^ The GREAT for diabetes offered a more structured and comprehensive understanding of type-2 diabetes over four interactive group sessions, facilitated in a guiding style. Unfortunately, during COVID-19, it was not possible to bring groups of people with diabetes together and this initiative was put on hold.^3^

The team of people responsible for GREAT for diabetes considered a number of alternative approaches to assist people with PEC despite the pandemic. One such approach was the conversion of the educational content into a WhatsApp Chatbot that delivered 16 short audio messages, supported by pictures.^9^ Evaluation of the Chatbot showed that it was feasible and had a positive effect on lifestyle change. Such services have been shown to have a modest effect on glycaemic control in other settings.^10^

The team also took note of initiatives within the Global Alliance for Chronic Diseases to conduct virtual group education with the use of tablets.^11^ The team thought that it would be valuable to explore whether this was feasible in our context for people with diabetes. The aim of this study therefore was to evaluate the first pilot attempt to virtually conduct GREAT for diabetes with a group of patients using tablets and Zoom video conferencing software.

## Methods

### Study design

This was an exploratory, descriptive qualitative study using focus group interviews with the facilitators and patients.

### Setting

The study was based in a community day centre in the Northern-Tygerberg substructure of Cape Town. This primary care facility was part of a participatory action research project to introduce more comprehensive PEC for people with non-communicable diseases, including diabetes. Piloting of virtual GREAT for diabetes was possible as part of this project, particularly as all other activities had stopped because of the COVID-19 pandemic. At this facility, prior to COVID-19, the dietician had been trained to offer GREAT for diabetes face-to-face to groups of people with diabetes.

Patients came from uninsured communities on the Cape Flats, mostly lived in low-cost housing and spoke either Afrikaans or English. The community was low income with high rates of poverty, crime and violence. Smartphones were commonly used in the community, but access to laptops or tablets was thought to be much less common. The purchase of data was also an issue for many people.

### Group Empowerment and Training for diabetes

When delivered face-to-face GREAT for diabetes consisted of four sessions that tackled the following topics:

- Understanding your diabetes
- Making lifestyle changes
- Understanding your medication
- Avoiding complications

Sessions were meant to include 10–15 people with a facilitator who might be a health promotion officer, a nurse or a dietician. Sessions were designed to be interactive with a variety of card games, pictures and resources to engage participants. Facilitators were trained in a guiding style of communication that was collaborative (involving the participants), evocative (obtaining ideas and solutions from the group), respectful (respectful of people’s choices and control), empathic (acknowledging people’s perspectives and experiences) and structured (keeping the group focused on the topics). This style and associated communication skills were derived from motivational interviewing.^12^ The central structuring device for all the sessions was an exchange of information using the elicit-provide-elicit communication strategy. In this process, the first elicit asks the group what they know or want to know, relevant information is then provided and a final and elicit asks what they will do with the information in their own lives.

The team already had a Zoom licence, which enabled people to meet virtually and for the facilitators to share pictures or resource materials on the screen. The team went through the training manual and saw that it was possible to conduct sessions virtually using Zoom. The dietician at the primary care facility who was used to conducting sessions face-to-face also brought in two of her colleagues from another substructure to assist. All three dieticians were fully trained in offering GREAT for diabetes. The project purchased a number of tablets with inbuilt cell phone connectivity and data that would enable connection to the Zoom meeting. The research coordinator prepared screenshots to explain how to use the devices and join the Zoom meeting.

In this initial pilot study of virtual GREAT for diabetes, five people with type-2 diabetes were identified at the facility by the dietician and invited to participate. Patients came to the facility to collect the tablets and to receive instructions in how to use them. They practiced connecting via Zoom at the facility before going home. All four sessions were then held over a 2-week period.

### Selection of participants

The three dieticians that facilitated the four sessions were invited to participate in a focus group interview. The five people with diabetes were also invited to participate in a separate focus group interview.

### Data collection

Semi-structured focus group interviews were conducted with the help of an interview guide. The guide was designed to explore different aspects of the virtual experience including the content of the sessions, the resources used, the facilitation, any technical issues and whether the initiative could be sustainable.

An independent researcher from Stellenbosch University who was familiar with GREAT for diabetes, but not involved in the pilot, interviewed the dieticians. The research co-ordinator (J.C.) who set up the virtual pilot, but did not interact directly with the patients, interviewed the patients. Interviews with the dieticians and patients were conducted virtually in a Zoom meeting and recorded.

### Data analysis

The two audio-tapes were transcribed verbatim and analysed by R.J.M. and D.S., who were not involved directly in the pilot. Analysis was assisted by Atlas-ti and followed a simplified form of the framework method^13^ to identify key themes.

## Findings

The two focus group interviews were conducted with the three dieticians and five patients. All three dieticians were trained in GREAT for diabetes and had professional experience that ranged from 5 to 13 years. One was based at the index facility and the other two came from the neighbouring substructure. The characteristics of the patients are shown in Table 1.

**TABLE 1:** Characteristics of patients.

Patient	Gender	Age (years)	Time since diagnosis	Treatment
1	Male	57	8 months	Insulin and metformin
2	Female	27	3 years	Insulin and metformin
3	Female	35	9 months	Oral medication
4	Female	48	1 year	Insulin and metformin
5	Female	48	6 years	Insulin and metformin

### Patient education and counselling during COVID-19

Patients reported that there was no previous education or counselling on their diabetes during the COVID-19 pandemic. They were just told that they were diabetic, but no explanations were given. Doctors only prescribed the medication, appeared rushed and gave them a return date for their next appointment. Patient felt offended at the lack of communication and empathy:

‘I do not want to speak badly of the clinic or anything, but I have never received education, because there … when I see a doctor, the doctor is so fast, I’m the only one who probably spend only five minutes in the doctor’s office. So for me it’s I’m not getting that doctor patient vibe.’ (FGI Patients)

‘Because is just “Okay, here’s your prescription. What’s wrong?” … Now they say “Okay, your sugar is high”. Now he puts you on insulin again or they give you more pills. Finished. Then I’m out of that doctor’s office again. As fast as … he just gives me prescription then I go to chemist and get my stuff. So there’s nothing for me to say right now, there’s no training or education or anything I can get there from that doctor. He’s so quick to get me out of that office, I do not know if I stink or smell or anything.’ (FGI Patients)

Patients experienced the clinic as having too few staff to attend to them all. The experience was mechanistic. Patients were diagnosed, given medication and had bloods taken. Yet there were no explanations about the findings and diagnosis and every visit was a repeat of the first. They were not asked whether the medication worked for them or not. The experience of the clinic was perceived to be the same before and during COVID-19:

‘There are too few people helping. Many just want to try to … as we go now then we go … when we come in then they ask “any disease?” Then you say “no” then we go to the prep. From the prep we go to the trauma and from the trauma they do … they draw your blood, you go back then you go to the chemist and there they give you medication and then you go home. This is how it works.’ (FGI Patients)

### Delivery of content and use of resources

The dieticians reported that it was easy to deliver the same content via Zoom and that it was helpful to have the pictures ready to share on the screen. Although patients were given a pack of pictures and materials to take home and use during the sessions this did not seem to work well. They felt that more graphics should be prepared to share on the screen, particularly for the various card games. It was also necessary for the dieticians to facilitate from outside their own facilities to avoid interruptions:

‘I think it helped having to have the graphics and things like on a screen for them to, I think that helped a lot. Because you never know with the, with the lighting and all of that, if you have to have like show something in front of a screen, like this. So I don’t think that will work much. But it’s nice to having to share your screen.’ (FGI facilitators)

Patients found that the content of the online group sessions for diabetes was useful. The information on portion sizes was particularly useful as patients were not aware that the portion sizes and types of food were contributing to increased weight gain and increased glucose levels:

‘It has taught me a lot about what … what diabetes is about. It made me very conscious to look after myself and it’s a good experience because we did not know what diabetes is.’ (FGI Patients)

‘Especially like me who does not know about portion size and that type of thing. It’s also something new. And I believe if I eat a bowl of food tonight, I do not like plates because plates look so big, the bowl looks small when you scoop in, then it is not a big portion.’ (FGI Patients)

Only one patient followed the exercise recommendations and the others felt they were not ready. Some patients experienced technical challenges with the tablets and did not recall all the content that was shared by the facilitators in the session on food groups. The time allocated was deemed sufficient for each session and the starting time was appropriate.

### Communication style and interaction

In the virtual setting, the facilitators were less able to read the group and judge levels of engagement and understanding. The group members were less responsive and interactive than in the face-to-face setting. The effect of this on the facilitators was that they tended to move through the material more quickly and to resort to a more directive style of communication. Nevertheless, they sensed that the patients were interested and excited to participate.

Facilitators thought that the sessions worked well with two facilitators to share the load and bring different personalities to the group:

‘There wasn’t a big difference in delivering the content, whether it was in face-to-face or virtual, there was not any difficulties having to, to relay the message. The only thing that stood out for me was having, I subconsciously, always thought about like, are they engaged? Are they still with me? Do they understand what I’m trying to say? I switch between languages when I see that they’re getting lost if I speak English, you know what I mean?’ (FGI facilitators)

‘I try to do the whole guiding style, always, but I couldn’t. There were times where I did the, what do you call it, the directive communication style? Because you will ask a question, and then you will wait, and then they won’t answer.’ (FGI facilitators)

Patients experienced the communication style as friendly, inviting and collaborative and felt able to ask questions and seek help within the group. It was the first time they had been in a group setting, receiving diabetes education and could not compare the communication style with what they had previously not experienced. Patients felt like they were part of a family, and everyone had the freedom to speak and appreciated that the information was given at their level of understanding:

‘One feels comfortable asking questions and you get an answer. I asked a lot of things and I got answers to my stuff I asked for. I do not have to be afraid to have asked for help for my smoking. I was just being honest.’ (FGI Patients)

### Advantages of distance education and counselling

Patients found that doing the online group education in the comfort of their homes to be beneficial, as it allowed them to do household chores and consume food and drinks while listening. This, however, could have been distractive as patients were not always attentive and had to be reminded and prompted by the facilitator. The online sessions at home also cut out travelling costs and the time taken to commute and wait at the clinic. Some of the more introverted patients believed they would not have spoken in the group setting at the clinic, yet felt comfortable to share and speak using the online platform as it was less intimidating:

‘If I had been to the Clinic, I could not make myself a cup of coffee. I could not have hung up my laundry. I cannot sit and eat my porridge. This is something much more comfortable. I could have gotten up quickly and quickly turned on the coffee … the kettle. You do not actually lose anything as you sit here now. And now I have to walk to the Clinic or walk or drive or whatever you do, which is more comfortable in your house.’ (FGI Patients)

### Concerns for safety of people and equipment

All the facilitators felt uncomfortable to take responsibility for the tablets. They were worried about storing the equipment at the facility and giving it to patients. Given the levels of poverty and crime in the community served, the facilitators expected the tablets to disappear. They were also worried that their offices might be targeted for theft. They commented that all the other staff and managers in the facility were highly sceptical that the equipment would be returned. When they met with the patients, they handed out the tablets in a private place and disguised them as books. Patients were surprised to be trusted with the equipment and a little concerned about whether this would also make them targets for crime. Out of the five tablets issues, four were returned immediately, and only one patient had to be followed up:

‘I wouldn’t want that responsibility on myself having to issue electronics to patients, and not knowing if I’m going to get it back. So that is a stress on another level. And I, and I would not want that stress.’ (FGI facilitators)

‘So and the negativity from the staff and everyone told me, there wasn’t I don’t think there was one positive staff member that would be like, it will work out, everyone thought that the patients are going to steal the tablets.’ (FGI facilitators)

Patients expressed their concerns that the tablets would get lost or stolen or people would sign up with the wrong intention of taking the tablet and not to receive the education. Patients expressed concern at the cost of data if they had to provide this themselves. However, when given data on the tablets, there was a risk that family and friends would use it, if there was no restriction:

‘Then Samantha’s child is on drugs, then he takes the tablet. He does not know what the reason for that thing which is laying there.’ (FGI Patients)

### Technical issues

Facilitators felt a bit intimidated at first, but the experience of using the tablets was not a problem for them and they did not need any extra training. Despite the training given to patients at the facility and the manuals with screenshots showing a step-by-step approach, it took time to get everybody connected, visible and audible. The older patients struggled more. They commented that it was necessary to have an additional person to support the technical issues and glitches so that the facilitator could focus on the education. One person lost connectivity in one session and could not re-connect. After the initial issues with connecting, the participants mostly only experienced minor technical issues such as switching the audio on and off:

‘I think we took about 40 minutes, just to get everyone on and can you hear me and can you and can you, can I hear you, you know all those technical issues and thank goodness we had an IT [*information technology*] guy in the background, because I don’t know, because we are not, we just know how to operate cell phones, we don’t know the ins and outs of Zoom and all of that. So it helps having that IT person trying to sort out the logistical issues before we could actually start the actual session.’ (FGI facilitators)

Patients expressed gratitude for the training they received on how to use the tablets and the follow-up calls to help them with technical matters:

‘Maurice thank you very much for all your help and that you called me also to teach me how to turn on the tablet, because there I struggled a bit for the sound, but I appreciate everything and I have very nice … I was quite well taught. Thank you so much Maurice and doctor.’ (FGI Patients)

### The future of virtual patient education and counselling

On the one hand, facilitators were surprised that the virtual sessions worked, but on the other hand, remained skeptical about taking this to scale in the public sector. To work with multiple groups a large amount of equipment would be needed. Patients could use smart cell phones, but the screen might be too small to enable engagement and data might be an issue when using their own devices. They felt that managers were not supportive of virtual approaches and their buy-in would be essential:

‘I think there is a place and like I said in one of the previous videos, initially, I was like, I don’t think it’s going to work. But now that we did it the first time, and I see the value and the way we could manage it. But I think in order to take it further, like you definitely going to needs support from your managers.’ (FGI facilitators)

The one facilitator recommended that this virtual approach to GREAT for diabetes be attempted in a more hybrid format. In her model, groups of patients could attend the wellness centres in the community and facilitators could lead sessions virtually via a laptop and large screen at these facilities:

‘But that is where the wellness sites come into play in the districts. So if we can roll out this great approach on a community level, where the patients can sit around in a room in front of a flat screen TV [*television*], and you have the facilitator on the other side of the screen.’ (FGI facilitators)

### Effects of the virtual patient education and counselling

Patients expressed sadness at having to hand in their tablets and the sessions coming to end, as they developed a sense of belonging via the online group but agreed to encourage each other to apply the lessons learnt via their cell phones. The sessions were helpful as the patients’ gained knowledge on how to manage their diabetes better. Patients felt they were able to control their diet more and understood the importance of cutting out sugary drinks and including more vegetables:

‘It has also taught me a lot. So I am very disobedient. I’m honest. I’m disobedient. If I want to eat then I want to eat. I want to eat sweets. I want to eat chocolates. I want to stay there like that craving. Now there is something new to learn, your portion size, you can have a nice meal but also a nice one without sugar. There are sweets without sugar. It’s sweet too, but it does not contain sugar. There’s a chocolate without sugar in it. That kind of stuff.’ (FGI Patients)

‘I changed my everything, eliminating soft drinks. I came to learn that your kidneys can pack up. I … stick with my water, my vegetables. If I cannot afford what I need, then I buy only one packet of vegetables maybe, a packet of carrots and that goes with everything in the foods, because I am afraid of the amputation. I do not want to go there.’ (FGI Patients)

## Discussion

It was possible to deliver GREAT for diabetes in virtual groups using an application such as Zoom and tablets. Even in the virtual space, participants continued to find the content, resource materials and guiding style of communication helpful. Participants were grateful for any attempt to provide them with education and counselling as this was lacking in their usual facility-based care. There were also some reported changes in lifestyle, particularly in terms of diet.

The technology allowed them to save money on travel and to save time attending the clinic for such education. Participants also felt more comfortable in their own homes, although they might be distracted. Participants struggled initially to become familiar with the tablets and Zoom and needed training, time and support. Everyone was concerned about the potential for the tablets to be stolen.

Another study conducted in the same context found that the facility-based staff was reluctant to introduce any innovation that would disrupt service delivery.^14^ By this they meant anything that would disrupt the flow of patients through the facility and hinder them getting through the workload during office hours. The perception that care was depersonalised, rushed, mechanistic and devoid of any education or counselling dovetails with this. In a resource-constrained context that is unlikely to employ additional human resources,^15^ the use of technology to engage patients directly might be a way of overcoming the barriers to patient education and counselling.

It was remarkably easy for facilitators to translate the face-to-face content and group processes into the Zoom environment. The one aspect that might need more attention was the use of the card games. Mobile-health (m-health) users in low-income and vulnerable communities appear to benefit from the use of multimedia and gamification,^16^ both of which are features of GREAT for diabetes. In this setting, it would be possible to make more use of video resources. There was also a suggestion that some people might find it easier to participate in a virtual context.

The use of tablets, provided by the university, is probably not a sustainable solution. The financial outlay to go to scale with providing such technology in the health services would be prohibitive, and there is a real risk of substantial loss through crime and even risks to personal safety. There is widespread access to smartphones in low-income communities in South Africa,^17^ and it is also better to engage people with familiar technology.^16^ Users need training for unfamiliar technology and experience more anxiety and uncertainty, all issues seen in this pilot.^16^ Future work should evaluate engagement with patients via their existing smartphones. The cost of data, however, would remain a problem although it might be possible to make such services free to the consumer through negotiation with cell phone companies. Another reason for choosing tablets was to make the screen larger and the pictures more visible, especially when people with diabetes may have limited vision.^18^

Another study in the same communities converted the GREAT for diabetes content into a WhatsApp Chatbot.^9^ This provided users with 16 brief audio messages and graphics on a smartphone. The evaluation showed that this was feasible and could go to scale in our context and had an effect on key behaviours as well as confidence.^9^ The disadvantage of the Chatbot was that interaction was minimal, and the motivational effect of group sharing and facilitation in a guiding style, derived from motivational interviewing,12 was absent. It could be that a range of educational options are needed, with face-to-face GREAT focused on newly diagnosed and uncontrolled patients,^7^ and virtual GREAT available to all, but directed more towards those that are better controlled.

Facilitators had also recommended a hybrid approach of facilitating groups at a distance in wellness centres. In this model, groups of patients would still need to gather face-to-face but could be led by a distant virtual facilitator. The advantage of such a model would be that one trained facilitator could have a greater reach, but the suggestion would need to be piloted and evaluated further. The use of community-based services to support chronic care is being actively explored.^19^ Community health workers may be able to provide basic health promotion, disease prevention and support adherence for people with chronic conditions.^19^ Their ability to provide in-depth PEC for a condition such as diabetes is, however, likely to be low, especially as health professionals themselves have limited knowledge.^20^ Community health worker teams and their parent non-government organisations do, however, also employ professional nurses and even dieticians. These professionals could offer group education in the community if this was part of their contract with the department of health.

This was a pilot study with one small group of patients that was intended to test whether the concept should be pursued further. The findings are therefore tentative and difficult to transfer to a wider context. The authors only explored the perspectives of those directly involved, and it may be helpful to elicit the opinions of facility management and those responsible for digital solutions and information technology in the Department of Health. Nationally there is support for developing M-health solutions in the health field.^21^ The Metro Health Services are currently developing their policy and strategies for digital health solutions and are not ready to commit to any innovations until this is clarified.

This study opens the way to consider how GREAT for diabetes could be offered virtually at a larger scale. Consideration should be given to use existing smartphone technology, and this might require a further study to explore its feasibility. The cost of data is a barrier that needs to be overcome. The use of information technology to extend the reach of trained facilitators might also be worth exploring.

## Conclusion

It is feasible to conduct the GREAT for diabetes programme virtually using tablets and Zoom meeting technology. The benefits of group interaction, sharing and motivation to change appeared to be retained. There were issues of a lack of familiarity with the technology and concerns that the hardware would be stolen in the community. Further studies should explore whether GREAT for diabetes can be successfully conducted using the existing patient-held smartphone devices.
